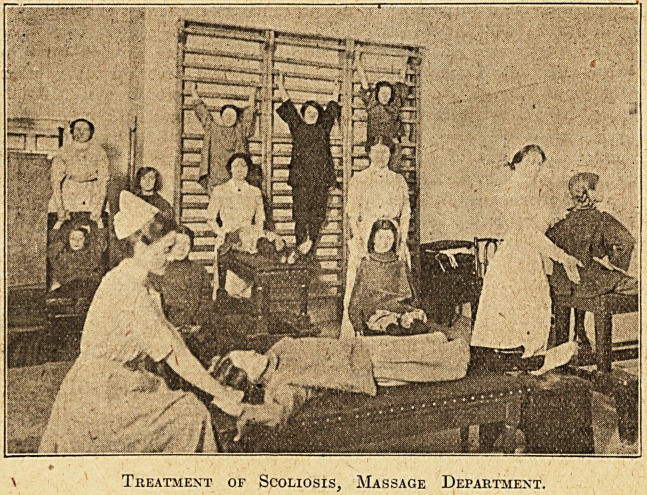# A School for Massage

**Published:** 1917-02-17

**Authors:** 


### A SCHOOL FOR MASSAGE.

### Developments at Guy's Hospital.

It is one of the direct consequences of the war that during the past two and a-half years the practice of massage has made greater progress than at any similar period of time since it was revived in this country and placed upon a proper and scientific basis. Perhaps it would not be too much to say that the war has done more for the advancement of this art than all the efforts of its advocates during the score of years that preceded the outbreak of hostilities. The reason is evident, for among the casualties of the many battlefields there are thousands and thousands of wounded and nerve-wracked soldiers whose pains are lessened and whose recovery is hastened by the employment of this method of treatment. In the early days of the war it was seen that all the trained masseuses available would be wanted for the work that lay ahead and that more and more would have to be trained if the full needs of the time were to be met. Fortunately the call for recruits was so quickly answered that many of the schools of massage had to be expanded to cope with the unprecedented rush of students. And it is only necessary to visit some of the large London schools to have ocular demonstration that, irrespective of class or age, women have come forward in large numbers, as in every other branch of activity, to aid the nation in its hour of need.

Treatment of Scoliosis, Massage Department.

It is, by the way, a curious fact that whilst this system is of great antiquity it is only in comparatively recent years that it has been accorded a place among our own recognised therapeutic methods. Paracelsus in 1591 " gave a description of methodical massage as practised by the Egyptians quite on modern lines," and the Swedish gymnastics introduced by Ling were based upon an old Chinese text-book. Its revival in Great Britain, at all events, or rather the placing of it upon a rational rather than an empirical basis, is largely due to the efforts of Dr. Weir Mitchell, who did much to secure public recognition of its utility, in combination with the " rest cure," as a treatment of hysterical and nervous conditions. The founding of the Incorporated Society of Trained Masseuses in 1894 and their constant supervision of training-schools has finally assured the status of the trained masseuse and eliminated the element of quackery.

There are now a number of schools throughout the country where massage is taught on scientific lines, some privately owned and others in connection with voluntary hospitals, and while some of the former compare favourably with the best of the institutional schools, it must be manifest that many advantages are to be derived from the association of the teaching of massage with the large selection of cases available for instruction at a large hospital.

One of the best examples of a school of this class is to be found at Guy's Hospital, and a representative of The Hospital, who recently paid a visit to this department at the busiest hour of the day, found a score or so of patients under treatment, and as many more in the waiting-room, an airy and comfortable structure, erected quite recently. Massage has been systematically practised at this hospital since 1888, but the growth of the department to its present important dimensions is comparatively recent. The school was opened to non-resident students in November 1914, and since then both the number of new patients and treatments given have increased enormously.

In order to afford accommodation for a considerable number of students and to give facilities for the treatment of as many cases as possible the department has recently been extended. Thus a waiting-room, already referred to, has been erected which is capable of holding a hundred patients, and the equipment of the school allows twenty students to work at one time. It is worthy of note that the necessary apparatus has been specially designed for the school and actually made in the works department of the hospital. Formerly the schoolroom and gymnasium were combined, but now there is a gymnasium 80 feet by 16 feet, a massage-room 40 feet by 16 feet for the students to practise on each other, and three classrooms, each to hold twenty-four students. About fifty students are now training for the different examinations of the Incorporated Society of Trained Masseuses, which are held every three months.

The older part of the school is situated on the ground floor of Clinical House, but the recently equipped part occupies the top floor of the southern half of Hunt's House, and is reached by an electric lift on the main staircase of the building. At first thought it occurs to the visitor that it may occasion some inconvenience to have the department so distinctly separated; but this is not really the case, for in practice it is found to be more convenient to have two distinct sections. The older part is devoted to the treatment of less serious cases, stiff joints, fractures, dislocations, and the like, and it is here that the students taking the elementary course are trained. In the newly equipped portion in Hunt's House more complicated cases are treated; various forms of nervous diseases, scoliosis, rheumatism, gout, and circulatory disorders, and it is here that the advanced pupils and those taking the course in Swedish remedial exercises receive instruction. The classrooms are well equipped with skeletons, anatomical models, and books of illustrative plates; and contain, in addition to the usual desks, abundant blackboard area, upon which the students are encouraged to draw their own diagrams, etc., the surest way of solving individual difficulties.

Furthermore, they have access to the incomparable collection of wax models in the Guy's Museum, and to prepared specimens in the dissecting-room, upon which weekly demonstrations are given by Mr. L. Bromley, F.R.C.S.

In addition there are lectures on anatomy four days a week, the syllabus of the examination being well covered. The weekly lectures on physiology are by Dr. Pembrey, a Reader in the University of London, and whilst dealing briefly with the whole subject have special regard to the underlying principles governing the administration of massage. Once a week a lecture is given on medical pathology, with clinical demonstrations on selected cases, by Dr. Poulton, M.R.C.P., assistant physician to the hospital, when the pupils are required to suggest forms of treatment. Similar demonstrations in the surgical branch are given by Mr. Trethowan, F.R.C.S., and attention is directed to the symptoms characterising the disease or injury and the programme of treatment fully described. Every morning, from 9 to 12, is devoted to practical work on the patients.

The department is under the charge of Miss H. S. Angove, to whom much of the information in this article is due, and under her is a staff of four qualified assistants, one of whom already holds a teacher's certificate and two more are preparing for it. The minimum duration of the course is six months, and for the certificate in Swedish remedial gymnastics an additional six months, though a year is strongly advised. Students may enrol at practically any time.

To show the large increase in the work of the school the following figures may be of interest.

During 1916 sixty-nine students entered for training in massage, eight failed to complete their training owing to various causes, and fifty-four gained certificates. Three, being minors, will be unable to compete till 1917; a similar number failed in part of the examination, and are re-entering in 1917. One only failed completely, and has since left the hospital. At the present time 350 patients are under treatment.

A tabulated comparison of the treatments in 1916 and 1915 will show the enormous increase in the cases dealt with:

| 1916. | | 1915. | |
|---|---|---|---|
| No. of New Cases. | | No. of New Cases. | |
| In-patients ... | ... 603 | In-patients ... | ... 385 |
| Out-patients ... | ... 1,744 | Out-patients ... | ... 1,112 |
| Total ... | ... 2,347 | Total ... | ... 1,497 |
| No. of Treatments. | | No. of Treatments. | |
| In-patients ... | ... 10,147 | In-patients ... | ... 5,471 |
| Out-patients ... | ... 39,312 | Out-patients ... | ... 19,751 |
| Total ... | ... 49,459 | Total ... | ... 25,222 |

Whether or no the present boom in massage is due solely to war conditions it is difficult to say. There is no doubt that many of those now qualifying will not find it necessary to gain a livelihood by that means, but will devote themselves to voluntary work for the wounded. Already at Guy's those who have gained their certificate are treating suitable cases in the special officers' section, and probably many others who have left are giving their services at V.A.D. hospitals in a similar manner. However this may be, one cannot look at the syllabus without being impressed by the diversity of subjects of which at least a slight knowledge is required, showing clearly that, contrary to public belief, the practice of massage does not depend merely upon manipulative and mechanical methods, but in its proper conception is based upon a knowledge of anatomy, physiology, and pathology. A visit to Guy's will convince anyone of the enthusiasm with which the students approach their work, and one feels assured that whether or no they choose this as their ultimate career in life they will, on finishing their course, have a clearer conception of the meanings of health and disease, a knowledge which, if spread broadcast throughout all classes, would result in that wisest form of prophylaxis—the prevention that is better than cure.

## Figures and Tables

**Figure f1:**